# Providence Insane Asylum

**Published:** 1862-12

**Authors:** 


### PROVIDENCE INSANE ASYLUM.

We have before called the attention of physicians to this Institution, its organization, location and general arrangements. It has now been in successful operation for something over one year, and received into its wards and private rooms quite a large number of patients, many of whom have been dismissed either entirely recovered, or more or less benefited, while other more obstinate or chronic cases remain, or have been dismissed, without important change. Among the list of entirely recovered, are some very interesting and important cases, the favorable termination of which has been a great gratification to all concerned. Of these cases it would be pleasant to speak in detail, but for obvious reasons we are at present unable to do so.

We have never been more favorably impressed with the internal condition and arrangement of any similar institution than with this. In a recent visit, we found some forty private rooms nicely furnished, containing all the comforts and luxuries of a desirable private residence, while the home-like appearance of the apartments at once impressed us with the conviction that it cannot be surpassed as a place of resort for the care and treatment of the unfortunate class of invalids it is designed to accommodate.

In Western New York and vicinity, it has so long been necessary to send insane patients to Eastern Asylums, that both the friends of such patients and their physicians had come to regard it as desirable, and even now, it may be supposed by some, not fully acquainted with the facts, that better accommodations can be obtained in the older or larger institutions.

If this is so, we desire as far as possible to correct this error, and assure any who may be interested, that nowhere can there be found an Asylum containing all the advantages of the larger and older institutions in greater degree than in the Providence Insane Asylum of Buffalo, while at the same time many objections which must necessarily pertain to crowded and over-patronized institutions are completely excluded.

Much is here done for the entertainment of the inmates, their tastes and fancies are gratified. The parlor is supplied with piano and the latest style of music for the amusement of those who have cultivation to enjoy it; and it is astonishing to see how much many insane patients will do for their own or other's entertainment. There is no appearance of restraint; patients mingle with, and become companions for each other, and it is quite amusing to hear one patient give the history and condition of another, telling you "she is a poor, crazy creature, and has been so for a long time," not once mistrusting that they are also "in the same boat."

There are other things which some of our readers may possibly desire to know concerning this Asylum, and so far as we are informed, it would be very pleasant to have all who desire it, made fully acquainted with every feature of the institution. It is in the care of the Sisters of Charity, whose constant and kindly ministrations are extended to all alike, regardless of rank or creed, and though God is worshipped by them through the forms of the Catholic Church, in their little private chapel, we give the assurance that this does in no way interfere with the fullest freedom of religious thought and opinion by any of the inmates, or detract in the slightest degree from its usefulness by making it sectarian in character. It is not a "Catholic Insane Asylum," as it is sometimes denominated by the uninformed, but an Insane Asylum in the care of the Sisters of Charity, whose attendance upon these patients has been abundantly demonstrated as superior in its influence upon the disordered mind, oftentimes calming the most violent, and subduing the hitherto unmanageable maniac. The gentle ministrations of a lady are often received with submission and thankfulness when the care of the best male attendant would be resisted with the greatest violence.

The proper management of the insane both hygienic and medicinal, is a subject with which, as physicians, we are too little familiar, and though much has been written and great improvements made in this direction within the last few years, still much remains to be learned and much of what is known has not yet been made, familiar to the general practitioner.

We hope to be able to gratify our readers with an occasional chapter upon these interesting subjects from some of our profession who have devoted time and study to the cultivation, and who are practically acquainted with all the more important phenomena attending the development and care of insanity.

